# Screening Strategy of Pancreatic Cancer in Patients with Diabetes Mellitus

**DOI:** 10.3390/diagnostics10080572

**Published:** 2020-08-08

**Authors:** Suguru Mizuno, Yousuke Nakai, Kazunaga Ishigaki, Kei Saito, Hiroki Oyama, Tsuyoshi Hamada, Yukari Suzuki, Akiyuki Inokuma, Sachiko Kanai, Kensaku Noguchi, Tatsuya Sato, Ryunosuke Hakuta, Tomotaka Saito, Naminatsu Takahara, Hirofumi Kogure, Hiroyuki Isayama, Kazuhiko Koike

**Affiliations:** 1 Department of Gastroenterology, Graduate School of Medicine, The University of Tokyo, Tokyo 113-8655, Japan; mizunos-int@h.u-tokyo.ac.jp (S.M.); ishigakika-int@h.u-tokyo.ac.jp (K.I.); saitoke-int@h.u-tokyo.ac.jp (K.S.); oyamah-int@h.u-tokyo.ac.jp (H.O.); hamadat-int@h.u-tokyo.ac.jp (T.H.); suzukiyu-int@h.u-tokyo.ac.jp (Y.S.); inokumaa-int@h.u-tokyo.ac.jp (A.I.); kanais-int@h.u-tokyo.ac.jp (S.K.); noguchik-int@h.u-tokyo.ac.jp (K.N.); satotat-int@h.u-tokyo.ac.jp (T.S.); hakutar-int@h.u-tokyo.ac.jp (R.H.); saitot-int@h.u-tokyo.ac.jp (T.S.); takaharan-int@h.u-tokyo.ac.jp (N.T.); kogureh-int@h.u-tokyo.ac.jp (H.K.); koike-1im@h.u-tokyo.ac.jp (K.K.); 2 Department of Endoscopy and Endoscopic Surgery, The University of Tokyo Hospital, Tokyo 113-8655, Japan; 3 Department of Gastroenterology, Graduate School of Medicine, Juntendo University, Tokyo 113-8431, Japan; h-isayama@juntendo.ac.jp

**Keywords:** pancreatic cancer, diabetes mellitus, risk factor, biomarker, screening

## Abstract

The incidence of pancreatic cancer (PCa) is increasing worldwide and has become one of the leading causes of cancer-related death. Screening for high risk populations is fundamental to overcome this intractable malignancy. Diabetes mellitus (DM) is classically known as a risk factor for PCa. Recently the reverse causality is in the spotlight, that is to say, DM is considered to be a manifestation of PCa. Numbers of epidemiological studies clarified that new-onset DM (≤2-year duration) was predominant in PCa patients and the relative risk for PCa inversely correlated with duration of DM. Among patients with new-onset DM, elder onset, weight loss, and rapid exacerbation of glycemic control were reported to be promising risk factors and signs, and the model was developed by combining these factors. Several pilot studies disclosed the possible utility of biomarkers to discriminate PCa-associated DM from type 2 DM. However, there is no reliable biomarkers to be used in the practice. We previously reported the application of a multivariate index for PCa based on the profile of plasma free amino acids (PFAAs) among diabetic patients. We are further investigating on the PFAA profile of PCa-associated DM, and it can be useful for developing the novel biomarker in the near future.

## 1. Introduction

The incidence of pancreatic cancer (PCa) is increasing worldwide, and PCa has become the 3rd leading cause of cancer-related death in the US [1] and the 4th in Japan [2]. Due to difficulty in diagnosing PCa in its early stage, only a limited proportion of patients can undergo curative resection. It is essential to select the high risk populations and conduct screening in asymptomatic individuals for improving the prognosis of PCa.

Diabetes mellitus (DM) is known as a risk factor for various malignancies: such as hepatocellular, breast, ovarian, endometrial, and gastrointestinal cancers; and total cancer incidence is reported to increase by 10% in diabetic patients [3]. DM is also recognized as a risk factor for PCa as in other cancers [4,5,6,7]. In addition, recent studies revealed that DM can develop as one of the symptoms of PCa [8,9]. Thus, DM, especially new-onset DM, is expected to be a clue to detect PCa in the early stage and improve the prognosis of this intractable malignancy.

However, the prevalence and the incidence of DM is too high, thus screening in all diabetic patients is ineffective in view of the cost-benefit balance. Further risk stratification for PCa among diabetic patients is warranted. In this review, we discuss the risk factors and biomarkers for PCa in diabetic patients.

## 2. New-Onset Diabetes Mellitus as a Clue for Early Diagnosis of Pancreatic Cancer

DM is a risk factor for PCa, and this classical causality was well-documented in previous studies. Everhart et al. reported a pooled relative risk (RR) of 2.1 (95% confidence interval (CI), 1.6–2.8) for PCa among diabetic patients in their meta-analysis of 9 cohort studies and 11 case-control studies [4]. Liao WC et al. recently carried out a systematic review of 9 studies (6 cohort studies and 3 case-control studies) and reported a pooled RR of 1.83 (95% CI, 1.50–2.24) [7]. Several studies also revealed a dose-response relationship between PCa incidence and fasting blood glucose [5,7,10]. Though the majority of these studies focused on type 2 DM, a few studies also documented that type 1 DM was a risk factor for PCa [11,12].

**Figure 1:**
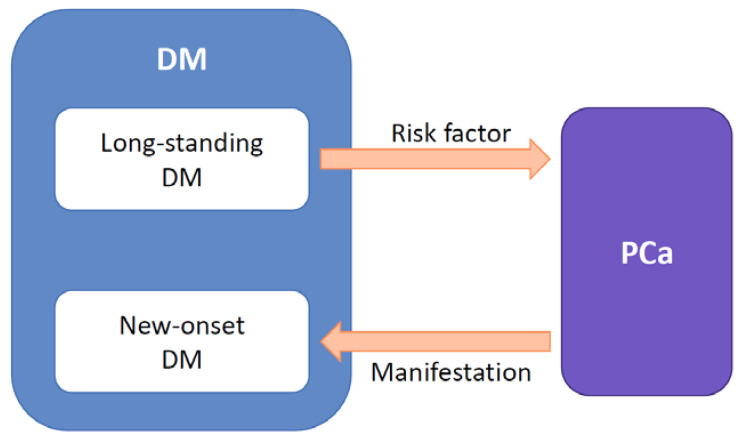
Bidirectional causality between diabetes mellitus (DM) and pancreatic cancer (PCa). Long-standing DM (> 2-year duration) is a risk factor for PCa, meanwhile new-onset DM (≤ 2-year duration) is a manifestation of PCa.

However, Chari ST et al. cast light on the reverse causality between PCa and DM. They revealed that new-onset DM (≤ 2-year duration) was predominant in patients with PCa and frequently resolved after surgical resection [13,14]. They also disclosed the increasing trend in fasting blood glucose before the diagnosis of PCa [8,13]. They speculated that new-onset DM was induced by the tumor, thus this “PCa-associated DM” can be classified as type 3c DM according to the classification proposed by the American Diabetes Association [15]. This strong association between PCa and new-onset DM was confirmed by many studies [16,17,18,19,20,21], and new-onset DM is now considered to be a manifestation of PCa (Figure 1). Among these studies, new-onset DM was defined as DM diagnosed within 2-3 years before PCa diagnosis.

**Figure 2:**
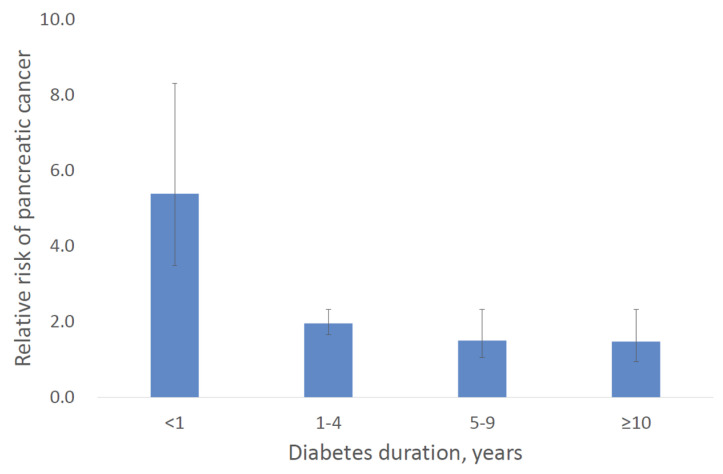
Relative risks of pancreatic cancer in patients with diabetes mellitus according to diabetes duration. The data are quoted from Ben Q et al. [9]. Bars indicate 95% confidence intervals.

Ben Q et al. provided the important evidence on the association between PCa risk and duration of DM [9]. In their meta-analysis, the highest RR of 5.38 (95% CI, 3.49–8.30) was reported in DM patients with short duration of 1 year or less. The RR for PCa gradually decreased according to diabetes duration: 1.95 (95% CI, 1.65–2.31) in 1–4 years, 1.49 (95% CI, 1.05–2.12) in 5–9 years, and 1.47 (95% CI, 0.94–2.31) in 10 years and more. Their results are summarized in Figure 2. Thus, new-onset DM can be a diagnostic clue to select high risk populations among diabetic patients.

As for high risk populations for PCa, intraductal papillary mucinous neoplasm (IPMN) of the pancreas is a well-established risk factor [22,23,24,25]. To date, a number of studies investigated the association between IPMN and DM. DM is prevalent in patients with IPMN [26,27], and Capurso G et al. reported an odds ratio (OR) of 1.79 (95% CI, 1.08–2.98) for IPMN in diabetic patients [26]. In addition, DM is reported to be associated with degree of dysplasia of IPMN [28,29,30]. Morales-Oyarvide V et al. reported that the OR for high grade dysplasia (HGD) and invasive carcinoma among diabetic patients was 2.02 (95% CI, 1.02–4.01) and 2.05 (95% CI, 1.08–3.87), respectively [28]. Thus, DM is a risk factor not only for a precursor lesion of PCa, but also for malignant transformation of the precursor. Meanwhile, new-onset DM is also reported to be associated with malignant IPMN [31,32,33,34,35]. Pergolini I et al. reported a high OR of 4.615 (95% CI, 1.423–14.698) for HGD/invasive cancer in patients with new-onset or worsening diabetes [31]. New-onset DM can be considered to be a manifestation of malignant transformation of IPMN, as in development of ductal adenocarcinoma.

We previously reported that PCa patients without symptoms, such as jaundice, pain, and appetite loss, in whom new-onset DM was a only clue to diagnosis of PCa had better prognosis than symptomatic patients (median survival time, 20.2 months vs. 10.2 months, *p* < 0.01) [19]. Pelaez-Luna M et al. reviewed computed tomography (CT) scans performed at PCa diagnosis and before diagnosis, and they reported that only 4 patients out of 13 had unresectable disease at the onset of DM [36]. Thus, screening for PCa among patients with new-onset DM can improve the prognosis.

However, PCa incidence within 3 years of meeting criteria for diabetes was reported to be only 0.85% by Chari ST et al. [37] and 0.25% by Munigala S et al. [38]. To date, there are a few studies attempting prospective screening for PCa in new-onset DM patients. Ogawa Y et al. reported that endoscopic retrograde pancreatography demonstrated PCa in 5 out of 36 diabetic patients (13.9%) with new-onset and onset after age 55 years [39]. Damiano J et al. reported that routine imaging, preferably by magnetic resonance imaging, in 115 patients with new-onset DM aged over 50 years detected 6 cases with PCa (5.2%) [40]. Illes D et al. performed abdominal ultrasonography in 115 patients with new-onset DM, then found 3 cases (2.6%) [41]. To establish effective screening in patients with new-onset DM, further selection using risk factors and biomarkers is warranted.

## 3. Risk Factors for Pancreatic Cancer in Patients with New-Onset Diabetes Mellitus

**Figure 3:**
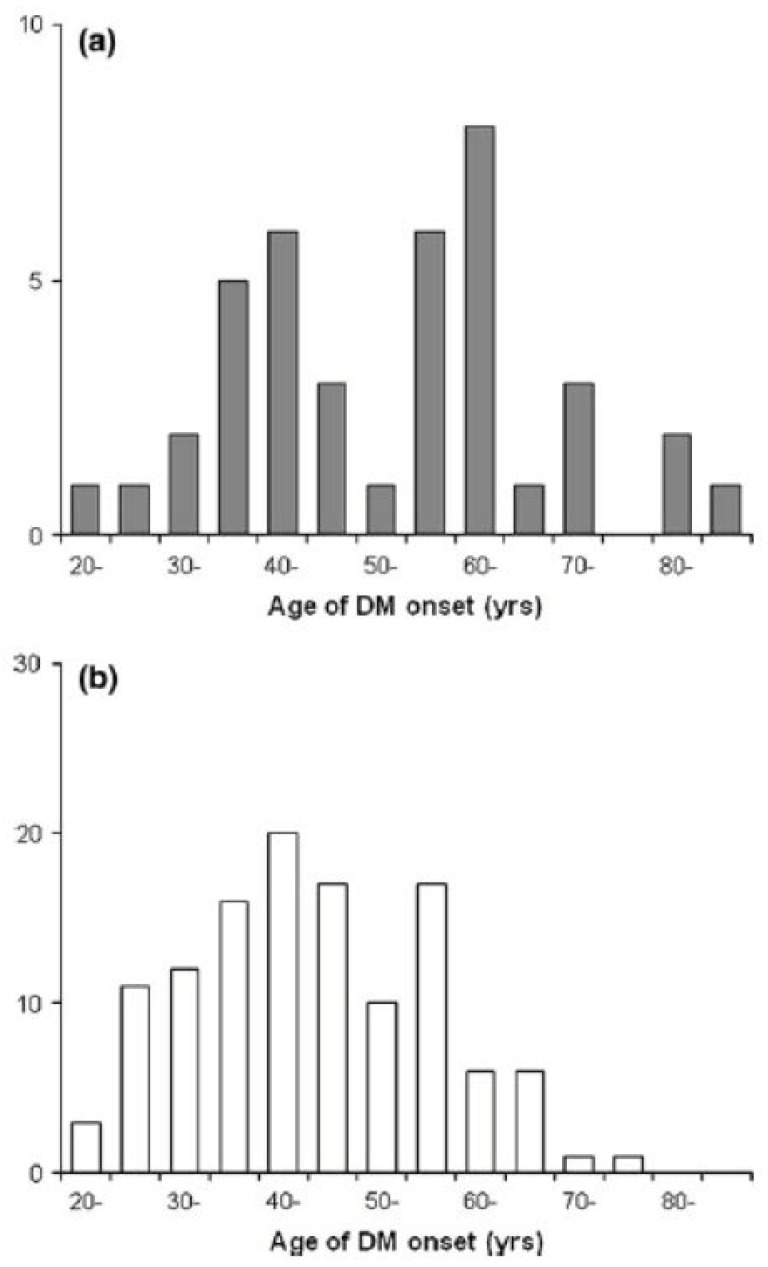
Age distribution of diabetes mellitus (DM) onset (**a**) in diabetic patients with pancreatic cancer and (**b**) in diabetic patients without pancreatic cancer. This figure is adopted from Mizuno S et al. [43].

In previous reports, several risk factors for and signs of PCa were investigated in patients with new-onset DM (Table 1). Elder onset of DM is a widely accepted risk factor [37,38,42,43], and the reported RR for PCa of individuals with DM onset age of 65 or elder was 2.01 (95% CI, 1.51–2.68) [38] and that of 70 or elder was 4.52 (95% CI, 1.61–12.74) [37]. We previously reported age distribution of DM onset comparing diabetic patients with PCa and those without. Among diabetic patients with PCa, there was a prominent peak between 55–65 years, and more than half were over 55 years old (Figure 3) [43]. Smoking and alcohol consumption are known as risk factors for PCa, and the synergic interaction of DM and these habits are reported [44,45]. Smoking is also well recognized as a risk factor for type 2 DM. Nicotine, the major component in cigarette smoke, is revealed to induce premature senescence of pancreatic β cells [46]. Meanwhile, nicotine is considered to reach the pancreas via the bloodstream or directly through the papilla Vater from the duodenum [47], then cause alteration in the signal transduction pathways and in the expression of protooncogene in pancreatic cells [48]. Thus, smoking is a risk factor of both PCa and DM.

**Figure 4:**
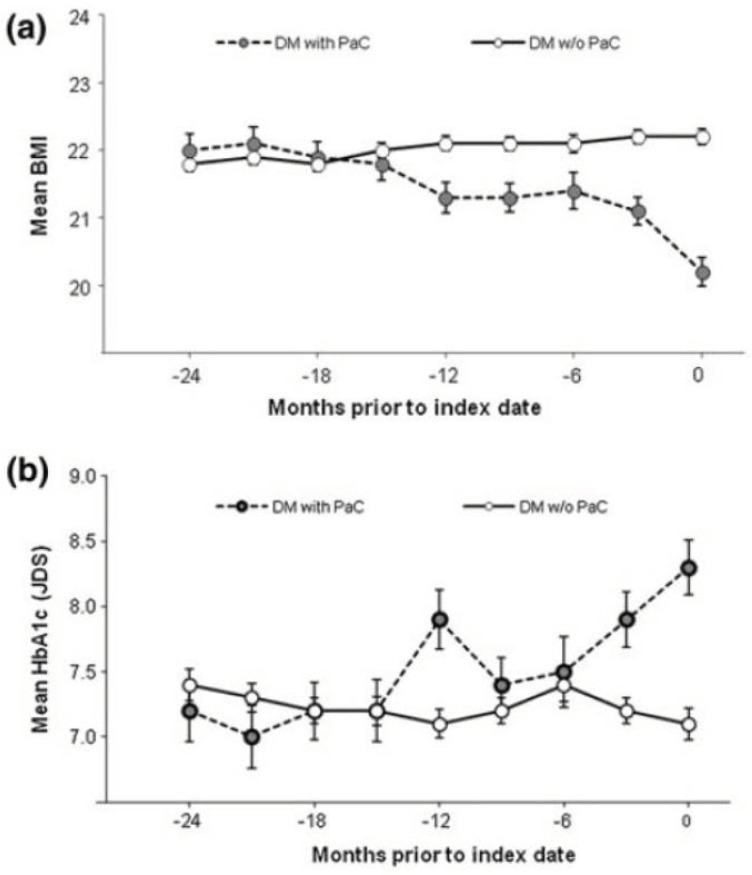
Temporal changes in (**a**) body mass index (BMI) and (**b**) hemoglobin A1c (HbA1c) in diabetic patients with pancreatic cancer (black circle with dashed line) and those without (white circle with solid line). This figure is adopted from Mizuno S et al. [43]. Bars indicate standard error of the mean. DM, diabetes mellitus; PCa, pancreatic cancer.

Body weight loss is a well-documented sign of PCa patients with DM [39,40,46,47,48]. Although body weight loss is a symptom also seen in type 2 DM subjects, Hart PA et al. reported significantly higher proportion of PCa-associated DM patients lost weight at onset of DM than type 2 DM patients (59% vs. 30%, *p* = 0.02) [49]. Mueller AM et al. calculated adjusted odds ratios (aORs) according to the degree of weight loss, and reported that aOR for weight loss by 10.0–14.9% was 3.58 (95%CI, 2.31–5.54) and that for loss by ≥ 15.0% was 4.56 (95% CI, 2.82–7.36), compared with stable weight [50]. Rapid exacerbation of glycemic control was also reported to be associated with increased risk of PCa. Huang BZ et al. reported more rapid increases in levels of glucose (37.47 mg/dL vs. 27.68 mg/dL, *p* < 0.01) and hemoglobin A1c (HbA1c) (1.39% vs. 0.86%, *p* < 0.001) in the month preceding the diagnosis of DM [51]. We previously demonstrated that simultaneous weight loss and HbA1c elevation were seen one year before the diagnosis of PCa among diabetic patients (Figure 4) [43]. The combination of signs can be helpful for early diagnosis of PCa.

## 4. Discrimination Models for Pancreatic Cancer in Patients with New-Onset Diabetes Mellitus

By using the combination of these risk factors and signs described above, enriching patients with new-onset DM for PCa screening is expected to be established. There are a few models reported to identify high risk individuals among patients with new-onset DM (Table 2).

Dong X et al. conducted a matched case-control study of 171 cases and 242 controls with new-onset DM and reported a prediction model which included 8 markers [52]. The area under the curve (AUC) of their model was up to 0.815, though the results were not validated.

Boursi B et al. conducted a retrospective cohort study of 109,385 patients with new-onset DM and reported a discrimination model which comprised 11 factors [53]. Their model provided an AUC of 0.82 and could reduce individuals who would undergo definitive screening to only 6.19% when the predicted risk threshold for PCa screening was set at 1% over 3 years. They performed internal validation using a bootstrapping procedure and reported negligible optimism.

Sharma A et al. reported a more simple discrimination model which was called Enriching New-Onset Diabetes for Pancreatic Cancer (END-PAC) [54]. They conducted a retrospective cohort study of 1561 patients with new-onset DM and developed the END-PAC model which comprised only 3 factors: change in weight, change in blood glucose, and age at onset. The AUC of this model was 0.87. In the validation cohort, an END-PAC score of ≥ 3 identified PCa with a sensitivity of 78%, specificity of 82%, and enriched the PCa prevalence of 0.82% in the population-based cohort to 3.6% (4.4-fold) in END-PAC model-defined cohort. The authors recently reported a larger-scale validation among 13,947 patients with new-onset DM [55]. The AUC was 0.75, and the sensitivity, specificity, positive predictive value, and negative predictive value were 62.6%, 78.5%, 2.0%, and 99.7%, respectively.

## 5. Biomarkers for Pancreatic Cancer in Patients with Diabetes Mellitus

In addition to the risk stratification by using the combination of risk factors, development of biomarkers to discriminate PCa-associated DM, which is classified as type 3c DM, from type 2 DM is expected to be useful for the effective PCa screening. Less invasive measurement of biomarkers can be a second sieve to select individuals who should undergo invasive examinations, such as endoscopic ultrasonography (EUS) and contrast-enhanced CT. Several biomarkers are reported to be potent for diagnosing PCa-associated DM (Table 1).

### 5.1. Islet Amyloid Polypeptide

Permert J et al. reported that plasma concentration of islet amyloid polypeptide (IAPP), a hormonal factor secreted from the pancreatic beta cells, elevated in patients with PCa-associated DM, though that in patients with type 2 DM was normal or low [56]. IAPP has diabetogenic effects in vitro and in vivo, thus was considered to cause insulin resistance in PCa-associated DM. Chari ST et al. confirmed the elevation of IAPP among patients with PCa-associated DM in their study of larger sample size [57]. However, the diagnostic yield was not satisfactory, and the sensitivity was only 50%.

### 5.2. Soluble Receptor 2 of Tumor Necrosis Factor-α

C-reactive protein (CRP) is increased in PCa patients due to the systemic inflammatory response to the tumor, and tumor necrosis factor-α (TNF-α) is an upregulating factor of CRP. Grote VA et al. measured the prediagnostic concentrations of soluble TNF receptors (sTNF-Rs) [58]. They disclosed that PCa risk tended to increase with higher levels of sTNF-R2 and this association was stronger in diabetic individuals. The OR of PCa for a doubling in sTNF-R2 concentration was 4.76 (95% CI, 1.11–20.37) among diabetic individuals, whereas that among non-diabetic individuals was 1.12 (95% CI, 0.73–1.72).

### 5.3. Osteoprotegerin

Osteoprotegerin (OPG) is a soluble decoy receptor for TNF-related apoptosis inducing ligand (TRAIL), and belongs to the TNF receptor superfamily (TNFRSF). Shi W et al. conducted a meta-analysis on gene expression microarray datasets, and found that OPG was up-regulated in PCa tissues [59]. The serum level of OPG was elevated in patients with PCa-associated DM, and the receiver operating characteristic (ROC) curve revealed that serum OPG provided an AUC of 0.737 with a sensitivity of 68.0% and a specificity of 73.9% to distinguish PCa-associated DM from new-onset type 2DM.

### 5.4. Vanin-1

Huang H et al. explored specific genes using microarray analysis of peripheral blood samples, which were unique in patients with PCa-associated DM compared with type 2 DM, and reported that vanin-1 (VNN1), a pantetheinase which acts a key regulator in oxidative stress, was a candidate for a biomarker [60]. Their group later investigated the functional mechanisms of VNN1 in the pathogenesis of PCa-associated DM, and disclosed that overexpression of VNN1 in the tumor tissues decreased glutathione concentration, increased reactive oxygen species (ROS), then aggravated paraneoplastic islet dysfunction [61].

### 5.5. Matrix Metalloproteinase 9

Matrix metalloproteinase 9 (MMP9) is one of the zinc-dependent endopeptidase family, and is synthesized by both tumor and peritumoral stromal cells. The expression of MMP by pancreatic tumor cells is considered to be an early phenomenon, since it occurs not only advanced, but also in borderline tumors and non-invasive IPMN [62]. Microarray analysis by Huang H et al. disclosed that the combination of VNN1 and MMP9 was useful to discriminate PCa-associated DM from type 2 DM [60]. Moz S et al. reported an AUC of 0.886 by combining MMP9 and CA19-9 for discriminating PCa-associated DM from type 2 DM, though there was no significant difference compared with CA19-9 only (AUC, 0.866) [63].

### 5.6. Insulin-Like Growth Factor

Insulin-like growth factor (IGF) is a growth hormone-regulated polypeptide involved in both human development and the maintenance of normal function and homeostasis in most human cells, and accumulating evidence suggests that serum concentration of IGF may influence cancer risk. Suzuki H et al. reported that the polymorphic variants of IGF genes might serve as a susceptibility factor for PCa, and disclosed a high OR of 5.69 (95%CI, 2.63–12.3) by joint effect of the susceptible allele and diabetes [64].

### 5.7. Circulating RNA

Recently, circulating RNAs are emerging as non-invasive markers for detecting PCa [65]. Tumor cells release substantial amounts of RNA into the bloodstream that strongly resist RNases in the blood, and these circulating RNAs are upregulated in the serum and plasma of cancer patients. Dai X et al. reported a six-serum mircoRNA panel with an AUC of 0.887 to discriminate PCa-associated DM form type 2 DM [66].

### 5.8. Plasma Free Amino Acid Profile

Recently, metabolomic analysis using liquid chromatography-mass spectrometry is applied to various cancers and disclosed disease-specific profile of plasma free amino acids (PFAAs) [67,68]. Fukutake N et al. developed a multivariate index for PCa based on the PFAA profile of PCa patients compared with that of healthy controls [69], and we previously reported a comparable diagnostic yield of this PFAA index in individuals with DM and those without [70]. Among diabetic patients, the sensitivity was 66.7% and the specificity was 92.7%. The concentration of PFAAs is reported to be affected by DM [71,72], thus further investigation on the difference of the PFAA profile between PCa-associated DM and type 2 DM can be useful for effective PCa screening among patients with new-onset DM.

A number of studies reported the possible utility of the biomarkers of PCa-associated DM However, these studies were case-control study setting with a limited sample size, thus there is no reliable biomarker to discriminate PCa-associated DM and type 2 DM at present.

## 6. Conclusions

To overcome the dismal prognosis of PCa, effective screening for asymptomatic individuals is required to be established. DM is an early manifestation of PCa, thus DM can be a diagnostic clue for early detection of PCa. New-onset DM is the first sieve to enrich diabetic patients for PCa screening, and the combination of risk factors and biomarkers will be the second one. In the US, a prospective study to establish a new-onset diabetes cohort of 10,000 subjects 50 years or older (NOD study) is ongoing [73]. Further advance in the research on the risk factors and biomarkers for PCa in new-onset DM is expected.

## Figures and Tables

**Table 1:** Risk factors, early signs, and biomarkers for pancreatic cancer in patients with diabetes mellitus.

Risk Factor	New-Onset (≤2-Year Duration) Elder-Onset (≥65 Years)
Early sign	Body weight loss Rapid exacerbation of glycemic control
Biomarker	Islet amyloid polypeptide (IAP) Soluble receptor 2 of tumor necrosis factor-α (sTNF-αR2) Osteoprotegerin (OPG) Vanin-1 (VNN1) Matrix metalloproteinase 9 (MMP9) Insulin-like growth factor (IGF) Circulating RNA Plasma free amino acid profile

**Table 2:** Discrimination models for pancreatic cancer in patients with new-onset diabetes mellitus.

Author	Sample Size	Study Design	Factors	AUC
Dong X [52]	413	Matched case-control study	BMI, age of DM onset, HBV infection, T.Bil, ALT, Cre, APO-A1, WBC	0.82
Boursi B [53]	109,385	Retrospective cohort study	Age, BMI, change in BMI, smoking, use of PPI, anti-diabetic medications, HbA1c, cholesterol, Hb, Cre, ALP	0.82
Sharma A [54]	1561	Retrospective cohort study	Change in weight, change in blood glucose, age at DM onset	0.87

AUC, area under the curve; BMI, body mass index; DM, diabetes mellitus; HBV, hepatitis B virus; T.Bil, total bilirubin; ALT, alanine aminotransferase; Cre, creatinine; APO-A1, apolipoprotein-A1; WBC, white blood cell; PPI, proton pump inhibitors; HbA1c, hemoglobin A1c; Hb, hemoglobin; ALP, alkaline phosphatase.
